# Effect of a Pregnancy Lifestyle Intervention Embedded Into a Home Visiting Program on Child Neurodevelopment

**DOI:** 10.1155/jnme/5518243

**Published:** 2026-04-29

**Authors:** Sarah S. Farabi, Rosemary Bass, Amit Mathur, Cindy Schwarz, Candice Woolfolk, Richard Stein, Samuel Klein, Debra Haire-Joshu, Alison G. Cahill

**Affiliations:** ^1^ Office of Nursing Research, Barnes Jewish College Goldfarb School of Nursing, St. Louis, Missouri, USA; ^2^ Department of Medicine, Division of Nutritional Science & Obesity Medicine, Washington University in St. Louis School of Medicine, St. Louis, Missouri, USA, wustl.edu; ^3^ School of Public Health, Washington University in St. Louis, St. Louis, Missouri, USA, wustl.edu; ^4^ Division of Neonatal-Perinatal Medicine, Saint Louis University School of Medicine, St. Louis, Missouri, USA, slu.edu; ^5^ Department of Obstetrics and Gynecology, Washington University in St. Louis School of Medicine, St. Louis, Missouri, USA, wustl.edu; ^6^ Department of Women’s Health, Dell Medical School, University of Texas at Austin, Austin, Texas, USA, utexas.edu

## Abstract

Maternal obesity is associated with a higher risk of abnormal child neurodevelopment. The aim of this study was to determine the effect of a lifestyle intervention embedded into a home visiting program, Parents as Teachers (PAT), on 18‐month neurodevelopment in the offspring. Pregnant women (9–15 weeks) of African American Race, 18–45 years old, first‐trimester BMI 25.0–45.0 kg/m^2^, and socioeconomically disadvantaged (e.g., Medicaid status) were randomized to standard home visiting (PAT only) or home visiting with lifestyle weight management counseling (PAT+), which began after the baseline assessment in pregnancy and continued until 12 months postpartum. Cognitive, language, and motor development at 18 months was assessed using the Bayley III Scales of Infant Development. Neurodevelopment outcomes were compared between groups and between child sex using un‐paired *t*‐tests and ANCOVA. 200 children (105 PAT+/95 PAT only) completed the neurodevelopmental testing at 18 months. Cognitive, language, and motor composite scores did not differ between groups. Across groups, number of PAT visits was positively correlated with scores in the cognitive and language domains. Our findings suggest that a lifestyle weight management program which decreased gestational weight gain does not affect offspring neurodevelopmental outcomes at 18 months.

**Trial Registration:** ClinicalTrials.gov identifier: NCT01768793

## 1. Background

Excessive gestational weight gain (GWG) in women who are overweight/obese is associated with an increased risk of abnormal child neurodevelopment [1]. These abnormalities include attention deficit hyperactivity disorder [2, 3], autism spectrum disorder [4], and cognitive/intellectual impairment [5, 6]. Lifestyle interventions have been shown to be effective at reducing total GWG in women with overweight/obesity. However, the effects of these interventions on child neurodevelopment, specifically cognition and language, are not clear.

We recently completed a randomized controlled trial that included a home‐based lifestyle weight management program focused on nutrition and physical activity within an existing national home visiting program (Parents as Teachers, PAT) that demonstrated a reduced weekly and total GWG in socioeconomically disadvantaged (SED, based on Medicaid status) African‐American women with overweight/obesity [7, 8]. PAT is a national, nonprofit, parent education and home visiting organization that serves diverse families from pregnancy until the youngest child in the home enters kindergarten [9, 10].

The purpose of the present study was to determine whether our lifestyle intervention embedded into PAT (PAT+) improved early (18‐month) offspring cognition, assessed by the Bayley Scales of Infant Development, compared to the standard PAT curriculum (PAT‐only). We hypothesized that offspring in the PAT + group would have better cognitive scores at 18 months compared to the PAT‐only group.

### 1.1. Study Participants

This study was a planned analysis for a secondary outcome (cognitive composite score) of the original study conducted as part of the Life‐Moms consortium [11]. A total of 267 African American women who were 13.3 ± 1.7 (mean ± SD) weeks pregnant, 25.8 ± 5.0 years old, with a BMI of 32.3 ± 5.0 kg/m^2^, and SED (92% Medicaid) were enrolled in the study. Participants were recruited from obstetrical clinics between October 2012 and March 2016. Written informed consent was obtained from all participants prior to beginning study procedures. The study was approved by the Institutional Review Board of Washington University in St. Louis.

### 1.2. Study Procedures

Full details of the protocol have been previously published [7, 8]. Participants were randomly assigned to receive either the standard PAT home visiting curriculum alone (PAT‐only) or the standard PAT curriculum enriched with lifestyle weight management counseling (PAT+) [7]. Randomization occurred by a third‐party web‐based generated randomization scheme, ensuring that investigators could not influence or predict group assignment. Standard PAT curriculum includes development‐centered parenting support, education, and parent‐child interaction using a strengths‐based approach. The PAT + intervention included goal setting for appropriate GWG, regular self‐assessment of weight, education and reinforcement of healthy eating and physical activity habits, promotion of breastfeeding, observational learning through role‐play, and modifications to the home environment. Participants in both groups received 1 h home visits from parent educators every other week during pregnancy and every other month for 12 months after delivery. Parent educators recorded the number of visits with each mother from enrollment until 12‐months postpartum. Weight and height were measured to determine BMI at baseline (15 weeks gestation) and at 34–36 weeks gestation. Cognitive, language, and motor development were evaluated in offspring of the PAT‐only and PAT + groups by a developmental pediatrician and a child/family psychologist by using the Bayley Scales of Infant Development‐Third Edition (BSID‐III) [12] at 18 months after delivery. Composite scores for each domain (cognitive, language, and motor) have a mean of 100 (SD = 15) at the 50^th^ percentile, with scores < 85 (1 SD below the mean) considered to indicate impairment for each domain.

### 1.3. Statistical Analysis

The cognitive composite score was the primary outcome for the analysis, and all other outcomes were exploratory analyses. Baseline characteristics between treatment groups were compared using *t*‐tests for continuous variables and chi‐square or Fisher’s exact for categorical variables, as appropriate. Because offspring sex differed significantly between treatment groups, analyses of neurodevelopmental composite scores were performed using analysis of covariance (ANCOVA) to adjust for sex. Multivariable logistic regression was used to compare impairment scores. Spearman correlation was used to assess relationships between the number of PAT visits and GWG with neurodevelopmental outcomes for the whole sample. A sensitivity analysis was conducted to assess potential selection bias among participants who completed neurodevelopmental testing. Baseline characteristics were compared between those who completed testing and those who did not. Analyses were conducted using STATA 18BE (StataCorp, LLC).

## 2. Results

A total of 200 children (75%) from the cohort (95 PAT‐only and 105 PAT+) completed the neurodevelopmental testing at 18 months. Maternal characteristics are provided in Table 1, and child characteristics are described in Table 2. Sensitivity analyses revealed that maternal and child characteristics (e.g., age, GWG, pregnancy complications, infant age and weight, and prevalence of SGA and LGA) were not significantly different between those who received neurodevelopmental testing and those that did not (data not shown). Consistent with the main study findings [7], maternal age and GWG were lower in the PAT + group (Table 1). There was a significantly higher percentage of female children in the PAT + group than in the PAT‐only group (Table 2). Neurodevelopmental outcomes are reported in Table 3. Cognitive, language, and motor composite scores of the PAT‐only group were not different than scores in the PAT + groups after adjusting for child sex (Table 3). Further, the percent of children with scores < 85 (indicating impairment) did not differ between groups for cognitive, language, or motor domains (Table 3). Full ANCOVA and logistic regression models are provided in Supporting Tables 1 and 2.

**TABLE 1:** Maternal demographics and metabolic markers.

	PAT‐only	PAT+	*p* value
Maternal Characteristics			
Age, years	26.7 (5.2)	24.9 (4.7)	0.01
Complications			
HDP	21 (22.1)	25 (23.8)	0.78
Gestational diabetes	10 (9.5)	10 (10.5)	0.81
Preterm birth	8 (8.4)	14 (13.3)	0.27
NICU admission	13 (13.7)	17 (16.2)	0.62
Nulliparity	76 (80.0)	76 (72.3)	0.21
Cesarean section	39 (41.1)	42 (40.0)	0.88
# of PAT visits completed	18.6 (6.6)	19.0 (7.0)	0.70
Maternal Metabolic Markers			
Baseline BMI, kg/m^2^	32.1 (4.9)	33.0 (5.2)	0.22
Gestational Weight Gain, kg	8.9 (5.4)	7.2 (5.3)	**0.03**

*Note: p* values represent values from independent *t*‐tests or chi‐square analyses; data are mean (SD) or *n* (% of total). Bolded value indicates statistically significant *p* value (*p* < 0.05).

Abbreviations: HDP, hypertensive disorders of pregnancy; NICU, Neonatal Intensive Care Unit; PAT, Parents as Teachers.

**TABLE 2:** Child characteristics.

	PAT	PAT+	*p* value
Child sex, (M/F)	52/43	37/68	**0.01**
SGA at birth	11 (11.6)	9 (8.6)	0.48
LGA at birth	4 (4.1)	7 (6.5)	0.45
Child age at testing, months	19.2 (1.6)	18.8 (1.3)	0.13
Child weight at testing, kg	12.0 (3.0)	11.5 (1.5)	0.09

*Note: p* values represent values from independent *t*‐tests or chi‐square analyses; data are mean (SD) or *n* (% of total). Bolded values indicates statistically significant *p* value (*p* < 0.05).

Abbreviations: LGA, Large for gestational age; SGA, Small for gestational age.

**TABLE 3:** Neurodevelopmental results.

	PAT	PAT+	*p* value
Cognitive composite	92.8 (9.9)	93.5 (7.9)	0.85
Language composite	89.2 (14.0)	91.5 (9.9)	0.44
Motor composite	95.5 (8.3)	96.4 (8.4)	0.92
Cognitive impairment	15 (15.8)	11 (10.5)	0.60
Language impairment	26 (29.2)	18 (18.8)	0.23
Motor impairment	7 (7.6)	8 (8.3)	0.49

*Note: p* values represent values from ANCOVA (composite scores) or multivariable logistic regression (impairment) which controlled for child sex; data are mean (SD) or *n* (% of total).

In both the PAT‐only and PAT + groups, females scored higher than males in cognitive (95.3 ± 7.5 vs. 90.6 ± 9.8), language (92.6 ± 13.2 vs. 87.5 ± 9.7), and motor (97.9 ± 7.7 vs. 93.5 ± 8.5) domains (*p* < 0.001 for all comparisons).

The number of recorded PAT visits was positively correlated with cognitive (*ρ* = 0.27, *p* < 0.001) and language (*ρ* = 0.21, *p* = 0.005) domains, regardless of group assignment. Further, across groups, GWG was not correlated with any domain.

## 3. Discussion

Pregnancies complicated by obesity are associated with an increased risk of abnormal neurodevelopment in the child, but it remains unclear if interventions which minimize GWG have beneficial effects on child neurodevelopmental outcomes. We evaluated whether a lifestyle intervention embedded into a home visiting program in SED African‐American pregnant women with overweight/obesity improved 18 month neurodevelopment scores in their children. Our data demonstrate that the lifestyle intervention, which decreased GWG, did not improve neurodevelopmental scores at 18 months. Females scored better than males in all domains in both groups, and language and cognitive scores were positively correlated with number of PAT visits.

Our findings are consistent with Spies et al., who demonstrated an antenatal lifestyle intervention embedded into routine care did not affect neurodevelopmental outcomes in children at 3 years of age [13]. Importantly, the Spies et al. trial did not have a beneficial impact on GWG, which is different from our intervention which did reduce GWG [13]. Our findings differ from Crovetto et al., who reported, despite no difference in GWG, improved cognitive outcomes in 2‐year‐old children born to mothers treated with a Mediterranean diet started in mid‐pregnancy [14]. This suggests that dietary composition may play a more important role than GWG in influencing neurodevelopmental outcomes for offspring. It is important to note that our control intervention was the standard PAT curriculum which is an evidence‐based program to promote child development. Thus, a lack of difference in findings may be due to both groups receiving home visiting. In our study, females scored higher than males for all three neurodevelopmental domains. This finding supports previous literature suggesting that female children tend to score higher on neurodevelopmental testing and that neurodevelopmental disorders, such as autism, are more common in males than in females [15, 16]. Of note, the tool used in our study does not evaluate for attention deficit or behavioral problems which may manifest at school age.

Limitations of this study include that we had 25% attrition for our primary outcome of cognitive development. As noted in the results section, we did complete a sensitivity analysis and found no differences between the moms and children that came in for neurodevelopmental testing and those that did not. This was a planned secondary analysis from the original trial; the original trial was powered to detect changes in GWG between groups [7], thus a lack of significant findings may be due to the fact that this study was underpowered. Assessment of neurodevelopmental outcomes at 18 months is limited due to the fact that most neurodevelopmental disorders are diagnosed later in life, but we view this as a critical time point which can be a marker for future disorder development. Our finding that language and cognitive scores were significantly correlated with the number of PAT visits is subject to confounding and bias; it could be that more engaged families also have access to more resources or that families who are able to have more PAT visits are systematically different than those who cannot. We are unable to untangle these factors in our current study, but our findings do highlight that home visiting may play an important role in achieving neurodevelopmental milestones.

## 4. Conclusion

In summary, we found that a lifestyle intervention embedded in a home visiting program which reduced GWG in SED African American women with overweight/obesity did not improve neurodevelopmental outcomes in offspring. Dose of home visits from parent educators was positively associated with higher language and cognitive scores at 18 months, and females scored higher than males in all three neurocognitive domains. Our findings suggest that while mitigating GWG is important for maternal and child cardiovascular health, it may not play a strong role in influencing cognitive development. However, home‐visiting programs, such as PAT, are important in helping children to achieve neurocognitive milestones, particularly in the first 2 years of life.

## Funding

This study was supported by the NIH grants NIDDK 5U01DK094416, P30 DK056341 (Washington University Nutrition and Obesity Research Center), P30 DK020579 (Washington University Diabetes Research Center), and the Foundation at Barnes Jewish Hospital.

## Conflicts of Interest

The authors declare no conflicts of interest.

## Supporting Information

Supporting Table 1: Neurodevelopmental Composite Score ANCOVA Models; Supporting Table 2: Neurodevelopmental Impairment Logistic Regression Models.

## Supporting information


**Supporting Information** Additional supporting information can be found online in the Supporting Information section.

## Data Availability

The data that support the findings of this study are available from the corresponding author upon reasonable request.
