# Supplementary material for: Effect of a Pregnancy Lifestyle Intervention Embedded Into a Home Visiting Program on Child Neurodevelopment
Source: J Nutr Metab. 2026 Apr 29;2026:5518243. doi: 10.1155/jnme/5518243 (PMC13127098; doi:10.1155/jnme/5518243)
Supplement: Supplementary file 1 — Supporting Information Additional supporting information can be found online in the Supporting Information section. [file JNME-2026-5518243-s001.docx]

**Supplemental Table 1: Neurodevelopmental Composite Score ANCOVA Models**

| Cognitive Composite Score | | | | | |
| --- | --- | --- | --- | --- | --- |
|  | | | Obs=200  Root MSE=8.63  R-squared = 0.07  Adj R-squared =0.06 | | |
| Source | **Partial SS** | **df** | **MS** | **F** | **Prob>F** |
| Model | 1071.73 | 2 | 535.87 | 7.19 | 0.001 |
| Group | 2.64 | 1 | 2.64 | 0.04 | 0.85 |
| Sex | 1048.55 | 1 | 1048.55 | 14.07 | 0.0002 |
| Residual | 14680.27 | 197 | 74.52 |  |  |
| Total | 15752 | 199 | 79.16 |  |  |

| Language Composite Score | | | | | |
| --- | --- | --- | --- | --- | --- |
|  | | | Obs=185  Root MSE=11.86  R-squared = 0.05  Adj R-squared =0.04 | | |
| Source | **Partial SS** | **df** | **MS** | **F** | **Prob>F** |
| Model | 1281.01 | 2 | 640.5 | 4.56 | 0.012 |
| Group | 82.87 | 1 | 82.87 | 0.59 | 0.44 |
| Sex | 1055.54 | 1 | 1055.54 | 7.51 | 0.007 |
| Residual | 25589.94 | 182 | 140.60 |  |  |
| Total | 26870.95 | 184 | 146.04 |  |  |

| Motor Composite Score | | | | | |
| --- | --- | --- | --- | --- | --- |
|  | | | Obs=188  Root MSE=8.09  R-squared = 0.07  Adj R-squared =0.06 | | |
| Source | **Partial SS** | **df** | **MS** | **F** | **Prob>F** |
| Model | 879.46 | 2 | 439.73 | 6.72 | 0.002 |
| Group | 0.61 | 1 | 0.61 | 0.01 | 0.92 |
| Sex | 846.05 | 1 | 846.05 | 12.93 | 0.0004 |
| Residual | 12102.89 | 185 | 65.42 |  |  |
| Total | 12982.356 | 197 | 69.42 |  |  |

**Supplemental Table 2: Neurodevelopmental Impairment Logistic Regression Models**

| Cognitive Impairment | | | | | |
| --- | --- | --- | --- | --- | --- |
|  | | | Obs=200  LR chi2(2) = 10.29  Prob > chi2 = 0.006  Pseudo R2 = 0.067 | | |
| Cog_Impair | Odds ratio | Std. err. | z | P>\|z\| | [95% conf. interval] |
| Group | 0.79 | 0.35 | -0.52 | 0.60 | 0.33, 1.89 |
| Sex | 0.26 | 0.12 | -2.84 | 0.004 | 0.10, 0.66 |
| _cons | 1.45 | 1.21 | 0.44 | 0.66 | 0.28, 7.47 |

| Language Impairment | | | | | |
| --- | --- | --- | --- | --- | --- |
|  | | | Obs=185  LR chi2(2) = 9.63  Prob > chi2 = 0.008  Pseudo R2 = 0.048 | | |
| Lang_Impair | Odds ratio | Std. err. | z | P>\|z\| | [95% conf. interval] |
| Group | 0.65 | 0.23 | -1.21 | 0.23 | 0.32, 1.31 |
| Sex | 0.39 | 0.14 | -2.58 | 0.01 | 0.20, 0.80 |
| _cons | 2.43 | 1.71 | 1.26 | 0.21 | 0.61, 9.68 |

| Motor Impairment | | | | | |
| --- | --- | --- | --- | --- | --- |
|  | | | Obs=188  LR chi2(2) = 9.01  Prob > chi2 = 0.01  Pseudo R2 = 0.09 | | |
| Motor_Impair | Odds ratio | Std. err. | z | P>\|z\| | [95% conf. interval] |
| Group | 1.47 | 0.82 | 0.69 | 0.49 | 0.49, 4.40 |
| Sex | 0.17 | 0.11 | -2.67 | 0.01 | 0.04, 0.62 |
| _cons | 0.57 | 0.64 | -0.50 | 0.62 | 0.06, 5.17 |
